# SportsXbiodata: A web platform for integrative analysis of exercise-induced gene and protein expression

**DOI:** 10.1016/j.isci.2025.114403

**Published:** 2025-12-11

**Authors:** Zheng Li, Zhexue Xu, Xiaolin Li

**Affiliations:** 1 Sports Science and Technology, Guangzhou College of Applied Science and Technology School, Guangzhou 510000, China; 2 Department of Neurology, Dongfang Hospital, Beijing University of Chinese Medicine, Beijing 100078, China

**Keywords:** health sciences

## Abstract

Exercise can induce extensive molecular adaptations across multiple tissues; however, existing databases primarily focus on transcriptomic data and lack the ability to integrate multi-omics evidence and literature within a systems biology framework. To address this limitation, we developed SportsXbiodata, an open-access multi-omics integration platform that compiles 2,563 public multi-omics datasets and 3,600 exercise-related publications. The platform provides comprehensive functional modules for gene/protein/metabolite expression profiling, immune infiltration analysis, phenotype-gene correlation analysis, knowledge graph analysis, literature-based gene exploration, and exercise-drug homology analysis. It also supports data querying, visualization, and user-defined enrichment analysis for exercise-related datasets. SportsXbiodata is completely free and accessible without registration, allowing users to directly download standardized datasets and analysis results, thereby promoting transparency and reproducibility in scientific research.

## Highlights

• SportsXbiodata integrates 2,563 multi-omics datasets and 3,600 exercise-related studies
• The platform provides comprehensive modules for expression, immune, phenotype and analyses
• Visualization, GSEA, and multi-omics integration are supported

## Graphical abstract

**Figure:**
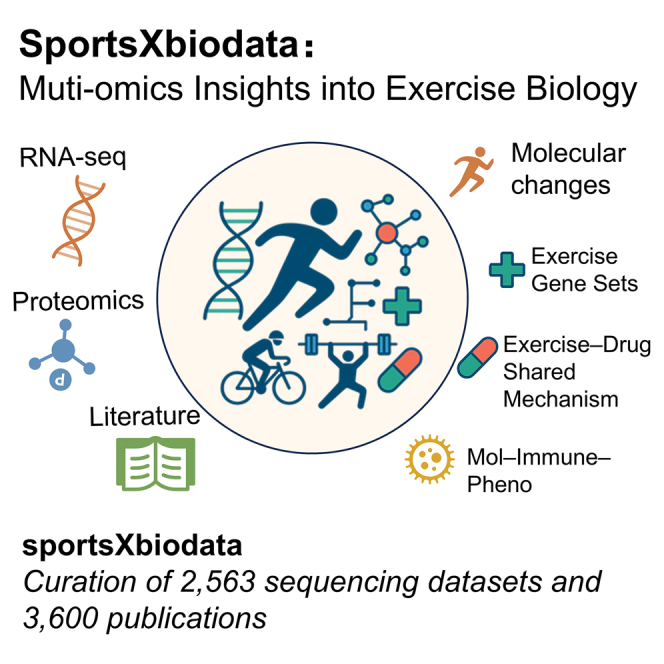

## Introduction

Life and exercise are inextricably linked, and studies throughout history have consistently confirmed the vital role of physical activity in maintaining health.^1^ In recent years, the rapid advancement of omics technologies and molecular biology has made it both possible and essential to investigate the systemic effects of exercise from a systems biology perspective.^2^ Extensive research has demonstrated that exercise triggers complex molecular and cellular responses, exerting multi-layered and tissue-specific biological functions within distinct microenvironments.^3^ However, as an intervention that profoundly impacts the entire body, the inherent complexity of exercise also presents significant challenges in the acquisition, integration, and analysis of related data. Therefore, an integrative perspective that combines multi-omics data with biological mechanisms is urgently needed to gain deeper insight into how exercise regulates physiological processes at the molecular level.

In recent years, several exercise-related database resources, such as GEPREP (gene expression profiles of RNA-seq-based exercise responses)^4^ and ExerGeneDB,^5^ have been developed, providing valuable information for elucidating the molecular mechanisms underlying exercise regulation. Among them, GEPREP offers relatively rich sample information but primarily focuses on transcriptomic data, which to some extent limits the potential for multi-layered integrative analyses and mechanistic exploration in exercise biology. With the rapid advancement of omics technologies, the widespread application and in-depth integration of multi-omics research approaches—such as proteomics and transcriptomics—have rendered it an urgent need and core direction in current exercise biology and related fields to dissect the molecular regulatory mechanisms mediated by exercise and unravel the laws governing their dynamic changes from a systems biology perspective. For example, the MoTrPAC (molecular transducers of physical activity consortium) project systematically explored and integrated the widespread impact of endurance exercise across multiple tissues and organs.^2^^,^^3^ Other studies have combined proteomic and transcriptomic analyses to identify key protein molecules involved in skeletal muscle growth regulation.^6^ These integrative multi-omics investigations have significantly expanded our understanding of the physiological effects of exercise and the complexity of its underlying molecular mechanisms.

However, current molecular biology data related to exercise remain scattered across various platforms and publications, lacking standardized formats and systematic integration, which severely hinders in-depth research on exercise mechanisms. There is an urgent need to systematically mine and integrate high-quality omics data and literature across diverse exercise contexts. Moreover, incorporating the effects of different exercise modalities on various disease states, along with their regulatory patterns at the molecular pathway level, will facilitate the elucidation of the diverse and specific biological effects of exercise in health maintenance and disease intervention. Therefore, the construction of a professional, systematic, and well-annotated integrative database for exercise-related molecular data has become a pressing and essential task in the current era of data-driven research.

We have developed a manually curated multi-omics database for exercise biology-SportsXbiodata (http://www.sportsxbiodata.cn/)—with the goal of systematically integrating a wide array of currently available exercise-related multi-omics datasets alongside high-quality literature in exercise molecular biology. The current version of SportsXbiodata contains 3,600 exercise-related publications and 2,426 multi-omics datasets, covering 12 tissue types and two species. In addition, SportsXbiodata offers a comprehensive collection of exercise-regulated molecular information, including differentially expressed genes, a knowledge graph of exercise-related genes, immune cell correlation analysis, phenotype association analysis, protein and phosphoprotein expression data, and multi-omics integration tools. Notably, SportsXbiodata allows users to upload their own omics data for in-depth analysis, such as automatic annotation of exercise-related genes and GSEA (gene set enrichment analysis). The platform also features a “drug-exercise homology” module, enabling users to identify potential exercise mimetics and explore shared regulatory mechanisms between pharmacological agents and aerobic exercise. In summary, SportsXbiodata aims to serve as an efficient and practical research platform that empowers users to explore the complex regulatory networks and causal mechanisms of exercise biology from a more systematic and global perspective.

## Results

### Overview of SportsXbiodata

**Figure 1:**
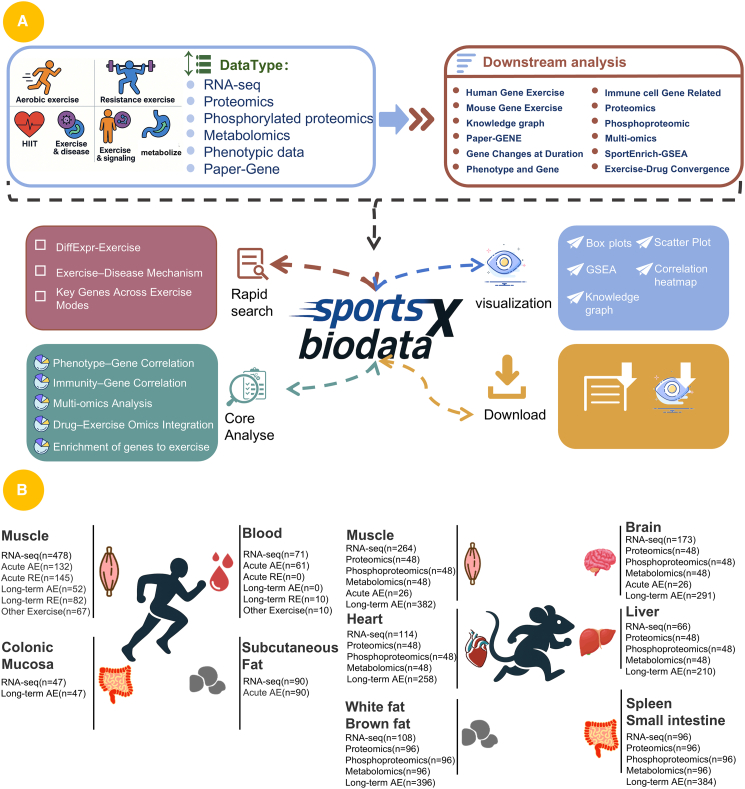
Platform content and construction (A) The SportsXbiodata platform systematically compiles a large collection of molecular biology data associated with various exercise conditions through manual curation. The datasets encompass multi-level information, including proteomics, transcriptomics, post-exercise phenotypic changes, and key exercise-responsive genes identified from published literature on exercise-related disease modulation. This comprehensive collection enables multi-dimensional analysis of abiological responses under diverse exercise modalities. In addition, SportsXbiodata offers a range of core analytical modules, including correlation analysis between phenotypes and gene expression, associations between immune parameters and gene expression, integrative multi-omics analysis related to aerobic exercise, comparative analysis of shared mechanisms between drugs and aerobic exercise, and enrichment analysis of exercise-related gene sets. The platform also supports a variety of visualization options and allows users to download analytical results, thereby meeting the diverse needs of researchers in data mining and interpretation. (B) Overview of RNA-seq data composition in SportsXbiodata. Among all collected datasets, RNA-seq was the predominant sequencing type. In mice, 1,973 samples were compiled, including 408 skeletal muscle, 258 heart muscle, 317 brain, 396 adipose, and 210 liver samples. For human data, a total of 686 samples were collected, comprising 193 acute resistance exercise samples, 478 skeletal muscle, 47 colonic mucosa, 90 subcutaneous fat, and 71 blood samples. Overall, muscle tissue was the most frequently analyzed, highlighting SportsXbiodata’s strength in supporting skeletal muscle-focused research. Detailed sample information is provided in Table S2.

The main framework and functionality of SportsXbiodata are illustrated in (Figure 1A). This platform supports a curated collection of multi-omics datasets generated under various exercise interventions, as well as exercise-related molecular biology data extracted from published literature. The data types include RNA-seq, proteomics, metabolomics, and phosphoproteomics, alongside key genes associated with exercise-induced responses and exercise-mediated disease modulation. All datasets have undergone standardized normalization procedures, followed by comprehensive downstream analyses. These analyses include differential expression analysis, correlation analysis between immune cell profiles and gene expression, correlation analysis between phenotypic traits and gene expression, investigation of shared mechanisms between exercise and pharmacological agents, and enrichment analysis of exercise-associated gene sets. Naming principles and dataset information are provided in Table S1. Collectively, SportsXbiodata offers a multidimensional data resource and analytical framework to support research in exercise biology.

### SportsXbiodata data type analysis

Among the data we collected, RNA-seq was the predominant type. Specifically, in mice, we compiled sequencing data from 1,973 samples. There were 408 skeletal muscle samples^7^^,^^8^^,^^9^^,^^10^^,^^11^^,^^12^^,^^13^^,^^14^^,^^15^, 258 heart muscle samples,^12^^,^^16^^,^^17^^,^^18^^,^^19^^,^^20^ 317 brain samples,^21^^,^^22^^,^^23^^,^^24^^,^^25^^,^^26^^,^^27^^,^^28^^,^^29^ 396 fat samples,^10^^,^^11^^,^^30^^,^^31^ and 210 liver samples.^11^^,^^32^ For human sequencing data, we collected 686 samples, of which acute resistance exercise samples were the most frequent, totaling 193 samples. There were 478 skeletal muscle samples,^33^^,^^34^^,^^35^^,^^36^^,^^37^^,^^38^^,^^39^^,^^40^^,^^41^^,^^42^ 47 colonic mucosa and,^43^90 subcutaneous fat,^44^ and 71 blood samples.^45^ Among all collected samples, muscle tissue was the most commonly analyzed, and RNA-seq emerged as the dominant sequencing technique. Details of the sample can be found in the supplementary table (Table S2). These observations suggest that SportsXbiodata possesses a distinct advantage for conducting research on skeletal muscle (Figure 1B).

During the literature collection process, we applied the keyword pattern “exercise AND” and systematically compiled over 119 research topics, which were subsequently classified into ten major categories based on thematic relevance. Among these, 11 topics were related to pure exercise modalities, encompassing aerobic exercise, resistance training, and high-intensity interval training. The neurological disorders category comprised 12 topics, such as Alzheimer’s disease, Parkinson’s disease, multiple sclerosis, and cognitive impairment. Metabolic diseases accounted for 13 topics, including diabetes, obesity, metabolic syndrome, and dyslipidemia. There were nine topics under cardiovascular diseases, such as hypertension, coronary artery disease, and heart failure. The respiratory diseases group contained eight topics, including asthma, chronic obstructive pulmonary disease, and pulmonary hypertension. Mental and psychological disorders involved eight topics, such as depression, anxiety, autism spectrum disorder, and bipolar disorder. The inflammation, immune system, and signaling pathways category included 12 topics, focusing on cytokines, NF-κB, Nrf2, autophagy, and apoptosis-related mechanisms. Gut, metabolic, and endocrine mechanisms comprised ten topics, addressing gut microbiota, mitochondrial biogenesis, and hormonal regulation. The musculoskeletal and motor system disorders group covered nine topics, including osteoporosis, osteoarthritis, and sarcopenia. Vascular diseases consisted of three topics, while pediatric developmental and learning disorders included five topics. This classification provides a systematic overview of current research directions in exercise-related interventions and offers a foundation for platform development and future studies. For detailed information, please refer to Table S3. The inclusion and exclusion criteria are shown in Table S4. Furthermore, we summarized 15 commonly used core terms in the field of exercise science and subjected them to more rigorous screening and standardization. On this basis, we systematically curated the direction of gene expression changes reported in the literature, along with the corresponding validation methods, and comprehensively documented key exercise-related parameters such as modality, frequency, and intensity. Finally, we integrated the genes associated with these terms into standardized functional enrichment terms for downstream enrichment analysis, please refer to Table S5.

### SportsXbiodata user interface

**Figure 2:**
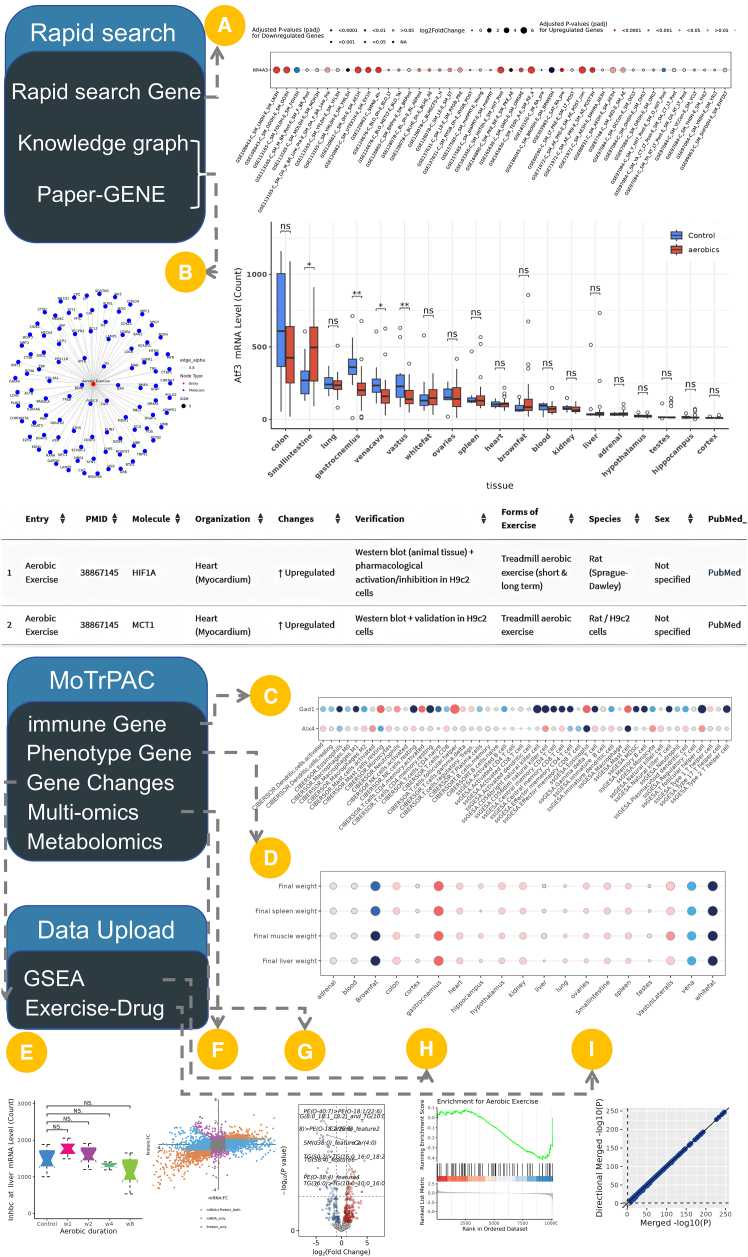
Main functions and applications of the SportsXbiodata platform (A) Users can flexibly explore the expression patterns of target genes across different exercise conditions and various organs or tissues. (B) The platform allows users to investigate diseases, signaling pathways, and core genes associated with exercise interventions under diverse exercise modalities. A knowledge graph can be generated, in which genes positioned closer to the central node typically represent current research hotspots. (C) Users can assess the correlation between target genes and various immune cell types to help infer potential immunological functions. (D) The platform supports correlation analysis between target genes and phenotypic traits (e.g., body weight and organ weight), enabling further biological interpretation. (E) Users can analyze the dynamic expression changes of target genes across different exercise stages (e.g., pre-exercise, various time points during or after exercise) within specific organs. (F) Differentially expressed molecules can be identified between selected groups, followed by integrative multi-omics analyses. (G) Users can utilize it to screen differential metabolites between different experimental groups. (H) Users can upload their own sequencing datasets to perform enrichment analysis of exercise-related core genes, aiding in functional annotation and mechanistic exploration. (I) The platform also supports user-uploaded drug-omics data to explore shared pathways and potential common mechanisms between exercise interventions and pharmacological treatments.

The SportsXbiodata platform provides users with multiple functions, including data querying, browsing, analysis, visualization, and download, enabling systematic exploration of information related to target genes, exercise modalities, and exercise-related disease interventions (Figure 2). In the rapid search module, the platform, leveraging exercise-associated samples, offers three efficient query modes: (1) exploring expression changes of target genes under different exercise conditions; (2) comparing pre- and post-exercise expression patterns of target genes across various tissues or organs (Figure 2A); and (3) determining whether target genes have been reported in exercise-intervention-related diseases and evaluating their research relevance, or alternatively performing reverse searches of core genes associated with specific diseases, signaling pathways, and exercise types. In addition, this module supports multi-dimensional association analysis, allowing users to freely select any two terms for joint analysis and identify key genes that simultaneously influence both terms (Figure 2B).

In the MoTrPAC module, the platform leverages multi-omics data from an 8-week aerobic exercise intervention in the MoTrPAC mouse cohort to enable more comprehensive and in-depth analyses. These include: correlation analysis between target genes and immune cell populations (Figure 2C); association analysis between target genes and phenotypic traits (e.g., body weight and organ weight), with an integrated sex-specific option that allows users to perform stratified analyses by sex (Figure 2D); visualization of tissue-specific temporal expression patterns of molecules across different exercise stages (Figure 2E); user-defined differential analysis of genes and proteins, as well as differential phosphorylation site analysis, followed by multi-omics integrative analysis (Figure 2F); and differential metabolomics analysis based on user-defined groups (Figure 2G). Together, these features enable a multidimensional dissection of the molecular mechanisms underlying exercise-induced adaptations.

In the data upload module, users can perform personalized analyses by uploading their own sequencing datasets. Two types of analyses are supported: (1) GSEA-based enrichment analysis of exercise-related core genes, with customizable selection of enrichment terms for functional annotation (Figure 2G) and (2) the exercise-drug module, which allows users to integrate drug-omics data with MoTrPAC datasets to identify potential shared pathways and common mechanisms between aerobic exercise and pharmacological interventions (Figure 2H).

### SportsXbiodata functional demonstration (I): Multi-omics integration reveals consistently regulated genes in exercise-responsive adipose tissue

**Figure 3:**
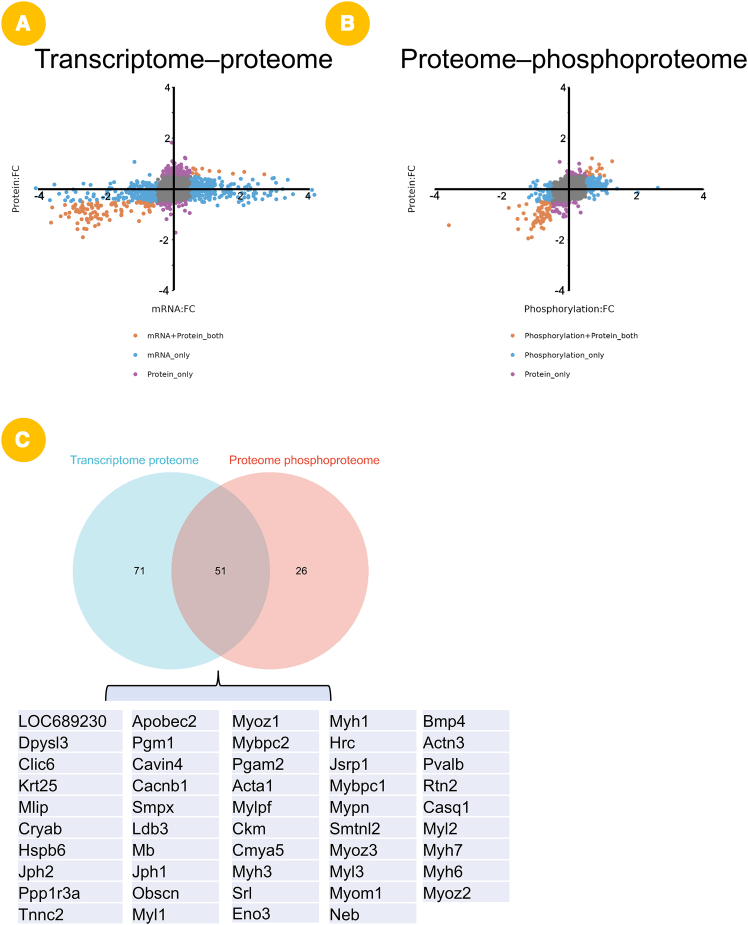
Case study 1: discovery of key exercise-responsive genes in adipose tissue (A) Combined transcriptomic and proteomic analysis (logFC ≥0.5) revealed genes with consistent mRNA and protein changes after exercise (*n* = 12). (B) Integration of proteomic and phosphoproteomic data identified molecules with concurrent protein abundance and phosphorylation changes. (C) Venn diagram intersection yielded 49 shared candidates.

To evaluate the practical utility of the SportsXbiodata platform in identifying key genes involved in exercise-induced physiological adaptations, we conducted a case study using adipose tissue as a model. First, we set the threshold to logFC = 0.5 and performed a combined analysis of transcriptomic and proteomic data to identify molecules whose mRNA expression and protein abundance were simultaneously and significantly altered in adipose tissue under exercise conditions, thereby pinpointing candidate genes with consistent regulatory trends (Figure 3A). Subsequently, we further integrated proteomic and phosphoproteomic data to identify key molecules exhibiting concurrent changes in protein abundance and post-translational modification (phosphorylation) during exercise (Figure 3B). Next, we applied a Venn diagram-based intersection analysis to the above two sets of co-regulated molecules and ultimately identified 49 candidate genes (Figure 3C). Notably, this set included well-characterized regulators of exercise-mediated adipose metabolism, such as BMP4^46^^,^^47^ as well as a substantial number of previously underexplored molecules in the field of exercise science.

### SportsXbiodata functional demonstration (II): Systematic exploration of atf3 in the context of exercise

To further validate the practical utility of the SportsXbiodata platform, we conducted a comprehensive case study centered on ATF3 (activating transcription factor 3). ATF3 is a representative molecule in exercise biology and has been extensively reported in the contexts of skeletal muscle satellite cell regulation and neuronal plasticity, and is widely recognized to be closely associated with exercise-induced stress responses and tissue remodeling.^48^^,^^49^ Therefore, selecting ATF3 as the target gene is not only biologically meaningful but also enables us to examine the platform’s capability to identify well-established exercise-related key regulators. By integrating its expression profiles, literature trends, and phenotype association analyses under exercise intervention, we systematically evaluated the potential role of ATF3 in exercise adaptation, thereby demonstrating the value of the SportsXbiodata platform in multidimensional elucidation of exercise-responsive core molecules.

**Figure 4:**
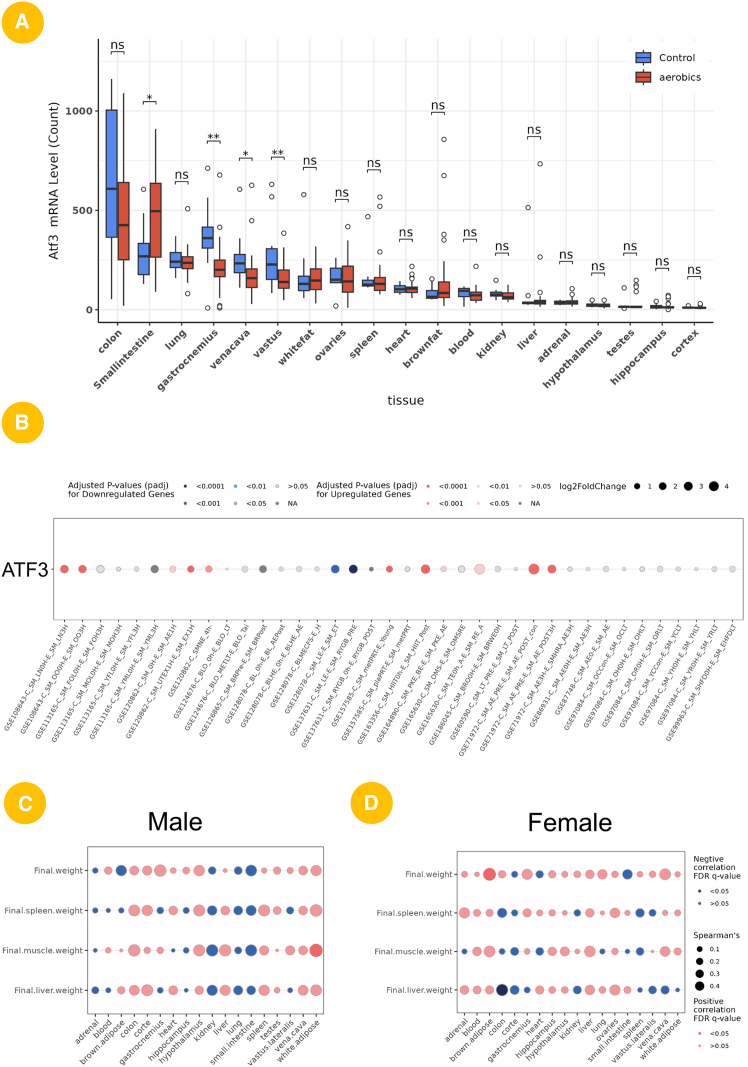
Case study 2: multi-dimensional exploration of a target gene in the context of exercise using the SportsXbiodata platform (A) Based on MoTrPAC data, the SportsXbiodata platform allows users to query the expression changes of ATF3 across different organs before and after exercise intervention. One-way analysis of variance (ANOVA) was used to assess statistical significance; ∗ indicates *p* < 0.05, ∗∗ indicates *p* < 0.001. (B) Utilizing GEO datasets, the platform enables analysis of ATF3 expression under various exercise modalities and intervention conditions. (C) Correlation between ATF3 expression in different organs and final phenotypic traits (e.g., body weight, liver weight, muscle weight, and spleen weight) in male mice. Dot color represents correlation direction (red = positive, blue = negative), dot size indicates Spearman’s correlation coefficient, and border style indicates FDR significance. (D) Female mice, enabling sex-specific comparison of ATF3-phenotype associations.

Based on transcriptomic data from the MoTrPAC project, we analyzed the expression changes of ATF3 across different tissues before and after aerobic exercise. The results showed that ATF3 was significantly upregulated in the colon (*p* < 0.05), small intestine (*p* < 0.01), lung (*p* < 0.01), and gastrocnemius muscle (*p* < 0.01), whereas no obvious changes were observed in other tissues, suggesting a tissue-specific responsiveness of ATF3 to exercise stimuli (Figure 4A). Notably, previous studies have primarily focused on the role of ATF3 in skeletal muscle, where it has been shown to participate in satellite cell regulation and muscle remodeling following exercise.^49^ However, the exercise-related functions of ATF3 in organs such as the small intestine, colon, and lung have been rarely reported, highlighting a potential research gap and suggesting that ATF3 may exert broader physiological roles beyond skeletal muscle in the context of exercise.

To evaluate the consistency of ATF3 expression changes under different experimental conditions, we further examined multiple GEO datasets encompassing various exercise types and durations. The dot plot illustrates both the direction (upregulation/downregulation) and statistical significance of ATF3 expression across datasets. ATF3 was frequently upregulated under resistance-related conditions, whereas it tended to be downregulated in endurance-related contexts (Figure 4B).

Finally, to investigate the physiological relevance of the observed molecular changes, we correlated ATF3 expression levels in each organ with final phenotypic traits such as body weight and organ mass (Figures 4C and 4D). The results revealed distinct correlation patterns between males (Figure 4C) and females (Figure 4D), suggesting that ATF3 may play sex-specific regulatory roles in linking exercise-induced molecular responses to physiological outcomes. It is worth noting that previous studies have reported that the biological functions of ATF3 are sex dependent^50^^,^^51^; therefore, we further examined its phenotype associations from a sex-specific perspective to more comprehensively elucidate its role in exercise.

### SportsXbiodata functional demonstration (Ⅲ): Application of SportsXbiodata in identifying shared pathways between drugs and exercise

**Figure 5:**
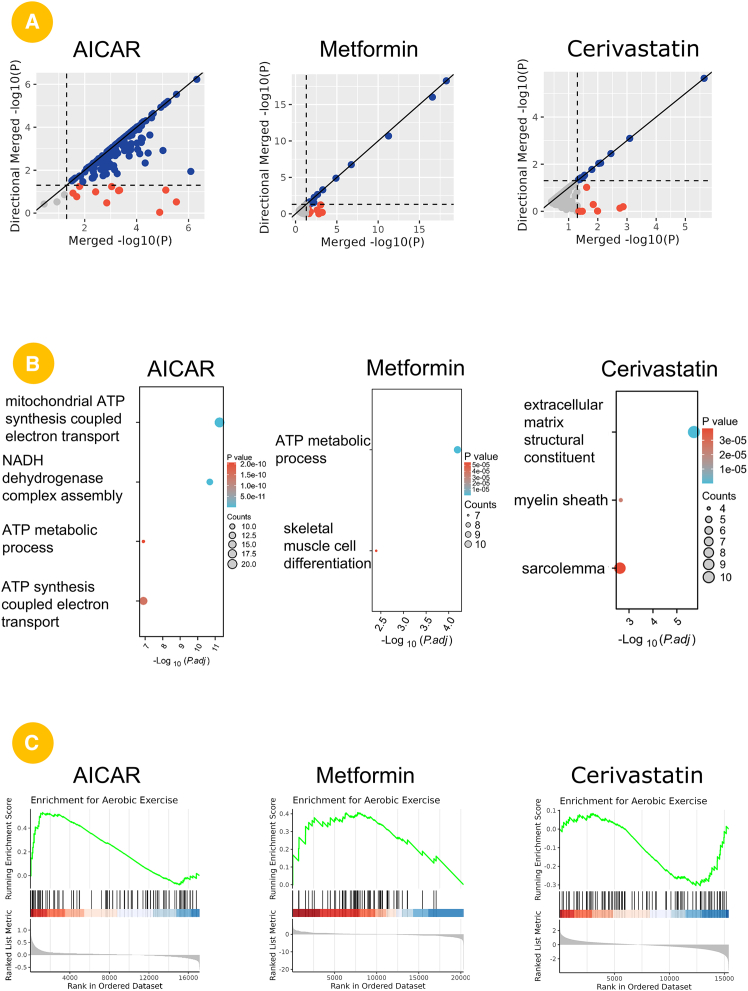
Case study 3: application of SportsXbiodata in identifying shared pathways between drugs and exercise (A) Directional significance analysis integrating transcriptomic and proteome profiles from aerobic exercise and drug perturbation. Metformin and AICAR exhibits a stronger overlap with exercise-responsive genes compared to cerivastatin. (B) GO enrichment of overlapping genes reveals that metformin-associated genes are enriched in ATP metabolic processes and skeletal muscle differentiation, while cerivastatin-associated genes are linked to extracellular matrix components and myelin-related terms. (C) GSEA shows metformin and AICAR yields a higher enrichment score for aerobic exercise-related gene signatures than cerivastatin.

To validate the functionality and practical utility of the SportsXbiodata platform in identifying molecules and pathways jointly regulated by drugs and exercise, we performed a systematic comparative analysis based on the transcriptomic and proteomic data of aerobic exercise and drug treatments (including metformin, cerivastatin, and AICAR). First, we applied directional significance testing to integrate the omics features of exercise and drug interventions, thereby identifying key pathways with shared regulatory effects. The scatterplot results show that, compared with cerivastatin, metformin and AICAR exhibit a stronger overall overlap with exercise-regulated genes, as evidenced by a greater number of genes with significant combined and directional *p* values (Figure 5A). This finding suggests that metformin and AICAR may share transcriptional characteristics with aerobic exercise more closely than cerivastatin.

We performed functional enrichment analysis on the overlapping gene sets between each drug and exercise. The results showed that metformin-associated genes were significantly enriched in biological processes such as ATP (adenosine triphosphate) metabolic process and skeletal muscle cell differentiation. In contrast, AICAR-associated genes were primarily enriched in mitochondrial ATP synthesis coupled electron transport, NADH dehydrogenase complex assembly, and ATP metabolic process. These processes are hallmark features of endurance exercise adaptation, suggesting that metformin and AICAR exhibit a high degree of functional consistency with exercise. By comparison, cerivastatin-associated genes were mainly enriched in terms related to the extracellular matrix, myelin sheath, and sarcolemma, indicating a functional profile that is clearly distinct from the other two drugs (Figure 5B).

To further assess the concordance between drug signatures and exercise-induced molecular profiles, we performed GSEA on exercise-related gene sets using the enrichment analysis module of the SportsXbiodata platform. The results showed that metformin and AICAR exhibited higher enrichment scores for aerobic exercise-related gene signatures than cerivastatin (Figure 5C).

## Discussion

The rapid advancement of omics sequencing technologies has greatly deepened our understanding of the systemic biological effects induced by exercise interventions. In recent years, several exercise-related databases have been developed to support molecular-level investigations into the effects of exercise across multiple organs and tissues, such as GEPREP and ExerGeneDB. Although these resources have played an important role in exercise science research, several limitations remain, including insufficient multi-omics data integration, incomplete literature-based gene annotation, the absence of drug-exercise homology analysis, and limited data visualization and interactivity. Specifically, GEPREP primarily focuses on providing sample-level metadata, while the data types in the aforementioned databases are still mainly based on RNA-seq and have not yet achieved comprehensive multi-layer omics integration. To address these gaps, we developed SportsXbiodata, a comprehensive platform that integrates high-quality literature evidence and multi-omics data. The platform aims to provide a more complete, visualizable, and interpretable molecular resource for exercise biology research, thereby promoting the systematic and refined development of this field. Importantly, SportsXbiodata is not intended to replace or compete with existing databases but rather to build upon the foundations established by GEPREP and ExerGeneDB. By extending their scope from single-omics to multi-omics integration and systematically enriching literature-based gene annotations, SportsXbiodata serves as a synergistic and complementary platform that further enhances the accessibility, comprehensiveness, and interpretability of exercise-related molecular data.

SportsXbiodata is not only a user-friendly database for querying, browsing, and visualizing exercise-related molecular biology data, but also serves as a valuable complement to existing resources in the field. Its main features are summarized as follows: (i) comprehensive multi-omics integration—SportsXbiodata systematically integrates exercise-associated omics data derived from multiple sequencing technologies, including RNA-seq, proteomics, and phosphoproteomics. The incorporation of data from diverse experimental platforms enables researchers to gain multidimensional insights into the systemic regulatory effects of exercise across different organs and tissues; (ii) high-quality literature support—the platform compiles a large collection of high-quality exercise-related publications and provides literature-based gene support scores and thematic classifications, thereby enhancing the biological interpretability and traceability of analytical results; (iii) drug-exercise homology analysis module—a built-in transcriptomic comparison module allows users to evaluate molecular similarities between drug perturbations and exercise responses, facilitating the identification of potential exercise-mimetic compounds and supporting drug repurposing and alternative intervention strategies; (iv) diverse interactive visualization tools—the platform offers various interactive visualization features, including enrichment plots, gene expression heatmaps, and phenotype correlation graphs. These tools are suitable for bioinformatics researchers seeking in-depth analysis as well as clinical and rehabilitation professionals aiming to efficiently extract key information; and (v) support for customized analysis—users can upload custom gene lists to rapidly retrieve expression profiles and perform functional enrichment analyses across multiple tissues and exercise conditions, thereby greatly improving analytical efficiency and enabling personalized research exploration. In summary, SportsXbiodata, with its depth of data integration, breadth of literature support, and high level of interactivity, establishes a comprehensive multi-omics research platform of both scientific and practical value for the study of exercise biology.

### Limitations of the study

Although SportsXbiodata provides a systematic and comprehensive research tool for elucidating the underlying biological mechanisms of exercise interventions, several aspects of the platform still warrant further refinement and optimization. First, the platform currently relies to some extent on heterogeneous datasets and lacks direct experimental validation, which may limit the expansion of its application scenarios and potentially affect the reliability of analytical outcomes. To address this issue, in the analytical workflow of this study, we did not directly merge raw expression matrices from different omics layers for joint modeling. Instead, we adopted a more robust and generalizable strategy: differential analyses were independently conducted within each omics layer (using the most appropriate statistical method for that data type), followed by cross-comparison and integrative analysis at the results level to identify key molecules exhibiting consistent regulatory trends or coordinated changes across multiple omics datasets. This strategy effectively reduces bias introduced by data heterogeneity, thereby enhancing the stability and reproducibility of the conclusions. Second, the current version of the platform does not yet include inter-organ interaction analysis. In future updates, we plan to systematically collect and integrate multi-organ omics data and to develop analytical modules for inter-organ regulation and signaling communication. These additions will enable the systematic exploration of dynamic molecular connections and cooperative regulatory mechanisms among organs, thereby providing a more comprehensive understanding of the systemic biological effects induced by exercise. In addition, SportsXbiodata currently focuses primarily on molecular-level analyses and has not yet incorporated exercise-related functional or physiological outcomes—such as dynamic changes in exercise performance, regulatory effects on health status, or responses of various health indicators. Accordingly, future work will further expand the analytical dimensions of the platform, emphasizing the exploration of association mechanisms between exercise and functional or physiological outcomes.

## Resource availability

### Lead contact

Requests for further information and resources should be directed to and will be fulfilled by the lead contact, Xiaolin Li (18810257677@163.com).

### Materials availability

This study did not generate new unique reagents.

### Data and code availability

• This study did not generate new datasets. All data analyzed in this study are publicly available from previously published sources and are summarized in the key resources table. The integrated results and visualizations are accessible through the SportsXbiodata web server (http://www.sportsxbiodata.cn/).
• All original code has been deposited at GitHub and is publicly available at https://github.com/lizheng199729/sportsXbiodata as of the date of publication.
• Any additional information required to reanalyze the data reported in this paper is available from the corresponding author upon reasonable request.

## Acknowledgments

This work was supported by the National High Level Chinese Medicine Hospital Clinical Research Funding (GSPQN2025-12).

## Author contributions

Conceptualization, Z.L.; methodology, Z.L.; investigation, Z.L., X.L., and Z.X.; writing – original draft, Z.L.; writing – review & editing, Z.X. and X.L.; resources, Z.X. and X.L.; supervision, Z.X. and X.L.

## Declaration of interests

The authors declare that they have no competing interests.

## Declaration of generative AI and AI-assisted technologies in the writing process

During the preparation of this work, the authors used ChatGPT 4.5 in order to improve the clarity and fluency of the English language. After using this tool or service, the authors reviewed and edited the content as needed and take full responsibility for the content of the publication.

## STAR★Methods

### Key resources table

**Table:** 

REAGENT or RESOURCE	SOURCE	IDENTIFIER
**Software and algorithms**		

R 4.3.2	R Core Team^52^	https://www.r-project.org
Shiny 1.9.1	Chang et al.^53^	https://github.com/rstudio/shiny
Shinydashboard 0.72	Chang^54^	https://cran.rstudio.com/web/packages/shinydashboard/index.html
DT 0.33	Xie et al.^55^	https://cran.rstudio.com/web/packages/DT/index.html
Xtable 1.8.4	Dahl et al.^56^	https://cran.rstudio.com/web/packages/xtable/index.html
ggplot2 3.5.1	Ginestet et al.^57^	https://cran.rstudio.com/web/packages/ggplot2/index.html
ggpubr 0.61	Kassambara^58^	https://cran.rstudio.com/web/packages/ggpubr/index.html
Limma 3.21	Ritchie et al.^59^	https://www.bioconductor.org/packages/release/bioc/html/limma.html
DESeq2 1.42	Love et al.^60^	https://bioconductor.org/packages/release/bioc/html/DESeq2.html
ActivePathways 2.05	Paczkowska et al.^61^	https://cran.r-project.org/web//packages/ActivePathways/index.html
clusterProfiler 4.10.1	Wu et al.^62^	https://cran.r-project.org/web//packages/ActivePathways/index.html
FastQC 0.11.0	Wingett et al.^63^	https://www.bioinformatics.babraham.ac.uk/projects/fastqc/
Fastp 0.23.4	Chen et al.^64^	https://pubmed.ncbi.nlm.nih.gov/30423086/
SortMeRNA 4.3.6	Kopylova et al.^65^	https://github.com/biocore/sortmerna
STAR 2.7.11	Dobin et al.^66^	https://github.com/alexdobin/STAR
SportXbiodata Source code	Zheng Li	https://github.com/lizheng199729/sportsXbiodata/tree/main
SportXbiodata Data	Zheng Li	http://www.sportsxbiodata.cn/

### Experimental model and study participant details

#### Public human and animal datasets

This study exclusively utilized publicly available human and mouse multi-omics datasets obtained from GEO, MoTrPAC, and ArrayExpress. No new human subjects, animals, plants, or cell lines were used.

#### Ethics for secondary data analysis

All original studies providing human or animal samples were approved by their respective institutional ethics committees. The present study performed only secondary analyses of de-identified public datasets and therefore required no additional ethical approval.

#### Human dataset characteristics

Human datasets from GEO and PubMed-curated literature included adult participants undergoing various exercise interventions. Available metadata included: age, sex, health status, and exercise modality/duration as reported in the original studies.

#### Animal dataset characteristics

Mouse datasets (MoTrPAC and GEO) included C57BL/6J mice of both sexes, with clearly documented exercise protocols (endurance, resistance, voluntary wheel running). Age and housing conditions followed each original study’s ARRIVE-compliant experimental design.

### Method details

#### Data collection and database contents

##### Collection of data from the GEO (gene expression Omnibus)

To collect gene expression data from the GEO database, we followed a series of standardized procedures to ensure the reliable retrieval of data from biological databases. Initially, we conducted a search in the GEO (https://www.ncbi.nlm.nih.gov/geo/) database using keywords such as “RNA (Ribonucleic Acid) sequencing,” “exercise,” and synonymous terms like “transcription,” and “transcriptome.” We restricted the dataset type to high-throughput sequencing, explicitly excluding matrix expression, chip data and non-coding RNA analysis. We retrieved 210 candidate datasets. From these, we further excluded those that included pre-existing disease conditions and studies with incomplete experimental information. In our study, we included a total of 26 human research projects.^33^^,^^35^^,^^37^^,^^38^^,^^40^^,^^41^^,^^67^^,^^68^^,^^69^ In the mouse exercise animal models, we collected data from 14 studies.

##### Collection of data from the MoTrPAC consortium

A portion of the mRNA data and all proteomic, phosphoproteomic, and metabolomic datasets used in this study were directly obtained from the publicly available MoTrPAC database. Specifically, the mRNA data were analyzed using the raw count matrices provided by the database, whereas the proteomic, phosphoproteomic, and metabolomic data were derived from the normalized datasets released by MoTrPAC, which had undergone quality control and correction procedures.

##### Literature collection and screening

We performed a systematic literature search in the PubMed database without time restrictions, using 120 exercise-related keywords that covered diverse exercise modalities (e.g., endurance, resistance), metabolic processes, and associated diseases (Table S6). For each keyword, up to 200 relevant publications were retrieved. The titles, abstracts, and, when necessary, full texts were manually screened and reviewed. Key information—including PubMed ID, species, sex, and gene names explicitly mentioned in the context of exercise—was extracted from each article.

To minimize heterogeneity across studies, we included only original research and well-defined review articles involving human or mouse subjects with clearly described exercise interventions. All gene names were standardized using controlled nomenclature systems (HGNC for human and MGI for mouse), and duplicate or ambiguous entries were manually removed. Exercise type, intervention duration, and physiological context were annotated according to a controlled vocabulary to ensure consistency across datasets.

Finally, curated gene names were categorized according to their associations with specific exercise modalities or metabolic pathways (Table S3), and the inclusion and exclusion criteria are summarized in Table S4. Moreover, we identified 15 core terms commonly used in exercise science and applied more stringent curation and normalization procedures to these entries. Based on this refined subset, we systematically compiled the reported directions of gene expression changes, associated validation methods, and key exercise parameters (modality, frequency, intensity). Genes linked to these terms were subsequently integrated into standardized functional enrichment categories for downstream analyses Table S5.

##### Handling data heterogeneity

To minimize the impact of data heterogeneity on analytical outcomes and to ensure the reliability of cross-study and cross-omics comparisons, this study implemented systematic control strategies throughout data collection, preprocessing, and downstream analysis.

First, during the data selection phase, all GEO datasets were included based on uniform inclusion criteria, retaining only high-throughput RNA sequencing studies with clearly defined exercise interventions. Literature data were also limited to original studies with well-documented intervention protocols, excluding high-level reviews. All gene symbols were standardized according to official nomenclature systems—HGNC for human and MGI for mouse.

Second, all transcriptomic data underwent a standardized processing pipeline, including quality control using FastQC and Fastp, alignment with STAR, quantification with featureCounts, and differential expression analysis using DESeq2. Proteomic data were normalized and modeled using the limma package to ensure consistency in analytical logic and statistical methodology across data sources.

#### Database construction

##### Rapid gene search

To ensure the reliability of our analysis results, we reanalyzed the data starting with a preliminary quality check using FastQC^63^ to evaluate metrics such as sequence quality, length distribution, and GC content. Following this, adapters and low-quality reads were removed using Fastp,^70^ and rRNA contamination was eliminated with SortMeRNA.^71^ Clean reads were then aligned to reference genomes (such as human hg38 (Genome Reference Consortium Human Build 38), or mouse mm10(Genome Reference Consortium Mouse Build 38)) using STAR,^72^ and alignment statistics were examined using SAMtools.^73^ The aligned data was used for transcriptome assembly with StringTie,^74^ and transcripts from multiple samples were merged using gffcompare. Next, gene or transcript expression levels were quantified using featureCounts,^75^ and expression normalization was performed by calculating FPKM (Fragments Per Kilobase of transcript per Million mapped fragments). To facilitate users in exploring the expression changes of genes of interest under different exercise contexts, we performed differential expression analysis using DESeq2^60^ across various group comparisons within the same dataset. The log2 fold change and adjusted *p*-values were extracted and compiled accordingly. For detailed group information, please refer to (Table S7). Data visualization was performed using the ggplot2 package in R.

To integrate gene expression patterns across multiple datasets with varying exercise conditions, we performed a meta-analysis based on differential expression results obtained using the DESeq2 package. For each gene, log_2_ fold changes and adjusted *p*-values were extracted from comparisons within each dataset. To capture the overall directionality of gene regulation, we calculated a direction score, defined as the difference between the number of datasets showing significant upregulation and those showing significant downregulation. A positive direction score indicates a gene is predominantly upregulated across datasets, whereas a negative score indicates predominant downregulation.

##### Knowledge graph

The knowledge graph layout was generated using the Fruchterman–Reingold force-directed algorithm, which positions nodes based on network connectivity such that genes co-occurring across multiple topics are naturally drawn toward the center.

##### Gene changes at different duration, proteomics,phosphoproteomic, metabolomics

In this module, transcriptomic, Metabolomics and proteomic datasets were directly obtained from the publicly available repository of the MoTrPAC, a large-scale, NIH-funded research initiative adhering to standardized experimental and quality control protocols. To ensure data reliability, we exclusively used datasets that passed MoTrPAC internal quality assessment pipelines. The processed data were subsequently analyzed and visualized using the ggplot2 package in R.

##### Phenotype and gene

In this module, we curated phenotypic data from the MoTrPAC dataset, including organ weights and terminal body weight of mice. Spearman rank correlation analysis was applied to evaluate monotonic relationships between gene expression levels and phenotypic traits, as this non-parametric method is robust to non-normal data distributions. The resulting correlations were visualized using the ggplot2 package in R to facilitate the interpretation of gene–phenotype associations.

Given that the sample size for each group was approximately 50, power analysis indicated that the correlation analysis had sufficient statistical power (≈0.8) to detect moderate-to-strong correlations (ρ ≥ 0.4) at a significance level of 0.05, but limited sensitivity for weak correlations (ρ < 0.3). Therefore, users are advised to focus on correlations with an absolute coefficient (|ρ|) of at least 0.4 to ensure reliable interpretation.

##### Immune cell gene related

In this module, immune cell activity and composition changes were estimated based on the publicly available MoTrPAC aerobic exercise transcriptomic datasets using two complementary computational approaches: ssGSEA (single-sample Gene Set Enrichment Analysis) and CIBERSORT.

For the ssGSEA analysis, we implemented the algorithm using the GSVA R package (method = “ssgsea”, kcdf = “Gaussian”, abs.ranking = TRUE). The input matrix consisted of normalized gene expression profiles, and the immune cell–related gene sets were imported from a curated.gmt file (getGmt(“immune cell.gmt”, geneIdType = SymbolIdentifier())). To ensure the robustness of the enrichment calculation, we set the minimum and maximum gene set sizes to 10 and 500, respectively (minSize = 10, maxSize = 500), thereby excluding extremely small or large gene sets that might lead to unstable enrichment scores. The resulting ssGSEA enrichment scores were normalized across samples using min–max scaling to allow cross-sample comparisons.

For the immune cell deconvolution, CIBERSORT was applied with 100 permutations (perm = 100) and quantile normalization enabled (QN = TRUE) to infer the relative proportions of 22 immune cell types from bulk expression data based on the validated LM22 reference signature matrix. The CIBERSORT-derived immune fractions were used to complement the ssGSEA results, providing a comprehensive overview of immune-related changes induced by aerobic exercise.

The Spearman correlation analysis was performed using all available samples from the MoTrPAC dataset, with approximately 75 transcriptomic samples per organ. The relatively large sample size ensures robust statistical power for correlation testing; however, even weak correlations may reach statistical significance. Therefore, users are advised to focus on correlations with an absolute coefficient (|ρ|) ≥ 0.3 to ensure biological relevance in interpretation.

##### Multi-omics

The data utilized in this study were directly downloaded from the publicly available MoTrPAC database. Differential expression analysis of mRNA data was performed using the DESeq2 package, which models count data based on the negative binomial distribution and provides robust estimates of fold changes and statistical significance. For proteomic data, differential expression was assessed using the limma package, which applies linear modeling and empirical Bayes moderation to improve statistical power in protein-level comparisons. The resulting data were visualized using ggplot2, which was employed to generate comprehensive scatterplots and other graphical representations.

##### SportEnrich-GSEA

This module was developed based on the R packages clusterProfiler and enrichplot, enabling robust Gene Set Enrichment Analysis (GSEA) and functional result visualization. The exercise-related gene sets used for enrichment were derived from a systematic PubMed literature mining workflow. Specifically, fifteen exercise-associated keywords covering diverse physiological and pathological contexts were used for retrieval. To reduce semantic heterogeneity and enhance biological relevance, only original research articles with clearly defined exercise interventions were retained. From these publications, gene names explicitly associated with exercise were manually extracted, standardized according to controlled nomenclature systems (HGNC for human and MGI for mouse), and annotated using a controlled vocabulary describing exercise type and physiological context. The finalized gene sets were then formatted into.gmt files and served as the input database for enrichment analysis.

Users can upload their own sequencing data, and the system ranks genes according to statistical metrics (e.g., log_2_ fold change or −log_10_ P-value). To control for variability in background gene distributions, the enrichment background is dynamically defined based on the complete set of genes provided by the user. GSEA is subsequently performed to evaluate the enrichment of exercise-related gene sets. Visualization outputs, including running enrichment score curves and ranked gene list plots, are generated to intuitively and informatively display the enrichment characteristics and distribution patterns of the gene sets.

##### Exercise-drug convergence

In this study, both proteomics and transcriptomics datasets were downloaded directly from the MoTrPAC database, encompassing multi-organ omics data from mice subjected to aerobic exercise intervention. The downloaded datasets included differential expression data from RNA-seq and proteomics.

Transcriptomics Differential Analysis: The DESeq2 R package was used to perform differential gene expression analysis on RNA-seq data, identifying significantly altered genes. DESeq2 utilizes a negative binomial distribution model to account for sequencing depth and biological variability, making it well-suited for small-sample RNA-seq analysis.

Proteomics Differential Analysis: The limma R package was applied to the proteomics data for differential protein analysis. Limma is based on linear modeling and empirical Bayes methods, suitable for both labeled and label-free quantitation proteomics data.

In this study, the ActivePathways R package was used to integrate the differential analysis results from aerobic exercise multi-omics (transcriptomics and proteomics) and drug-related omics. This method combines significance P-values across multiple omics layers to identify consistently enriched pathways, thereby revealing the comprehensive regulatory effects of aerobic exercise on molecular networks and its potential shared mechanisms with drug interventions.

##### Update and maintenance policy

To ensure the long-term usability and high reliability of the SportsXbiodata platform, we have established a systematic and sustainable update and maintenance strategy. Specifically, the literature data are routinely updated every three months, during which newly published exercise-related studies are continuously retrieved and manually curated to ensure that the platform consistently reflects the latest advances in the field. The multi-omics datasets are integrated every six months, incorporating publicly available transcriptomic, proteomic, and phosphoproteomic data with standardized processing and batch effect control, thereby expanding data coverage while maintaining analytical quality and consistency. In addition, we have implemented a user feedback–driven iterative mechanism, through which functional module optimization and bug fixes are updated in real time based on user experience.

#### Case study methodology

##### Case study 1

In Case Study 1, we utilized the Multi-Omics and Proteomics–Phosphoproteomics Integration modules of the SportsXbiodata database to analyze white adipose tissue data. The control group was defined as “Control.eight.weeks.Male.white.adipose”, and the experimental group as “Training.eight.weeks.Male.white.adipose.” A log_2_ fold change (logFC) threshold of 0.5. Overlapping molecules between datasets were subsequently identified using the VennDiagram R package for intersection analysis.

##### Case study 2

The ATF3 gene was initially examined using the “Rapid Search of Mouse Genes” module, and subsequently cross-validated through the “Rapid Search of Human Genes” module within the SportsXbiodata platform. Correlation analyses were then conducted in the Phenotype and Gene module, with the sex parameter restricted to Male or Female, in order to assess potential sex-specific regulatory patterns.

##### Case study 3

In this study, the transcriptomic data for AICAR were obtained from GSE50873, comparing skeletal muscle samples from control mice and those treated with AICAR for eight consecutive days. The Metformin dataset was derived from GSE167462, in which we compared gene expression in skeletal muscle between control mice with muscle atrophy and those receiving Metformin intervention. The Cerivastatin dataset originated from GSE4418, where we analyzed gastrocnemius muscle samples from untreated control mice and mice treated solely with Cerivastatin. Differentially expressed genes were identified using a threshold of |log_2_FC| > 0.5 and adjusted *p* value <0.05, and these genes were subsequently incorporated into the Exercise-Drug Convergence module for further analysis.

For functional annotation, GO enrichment analysis was conducted using the clusterProfiler package in R, focusing on the shared genes identified within the drug–exercise module. GSEA enrichment analysis was performed through the SportEnrich-GSEA module of the SportsXbiodata platform, utilizing the log_2_FC values of all genes to comprehensively elucidate the functional associations between drug intervention and exercise-induced molecular characteristics.

### Quantification and statistical analysis

Statistical analyses used: Differential expression: DESeq2, limma. Correlation analysis: Spearman rank correlation. GSEA: clusterProfiler + enrichplot.Multiple testing correction: Benjamini–Hochberg FDR. Statistical reporting: n = ample count per dataset or per organ. Significance: adj *p* < 0.05 unless otherwise noted. Visualization: ggplot2. All statistical parameters, including sample sizes, test types, effect sizes, and adjusted *p*-values, are reported in Table S8.

### Additional resources

SportsXbiodata platform: [http://www.sportsxbiodata.cn/].

GitHub code repository: [https://github.com/lizheng199729/sportsXbiodata].
